# Supplementary material for: Modular footwear that partially offsets downhill or uphill grades minimizes the metabolic cost of human walking
Source: R Soc Open Sci. 2020 Feb 5;7(2):191527. doi: 10.1098/rsos.191527 (PMC7062060; doi:10.1098/rsos.191527)
Supplement: Supplementary Figures and Table [file rsos191527supp1.pdf]

Electronic Supplementary Material

Title:

Modular footwear that partially offsets downhill or uphill grades minimizes the metabolic cost of human walking

Authors:

Antonellis P, Frederick CM, Gonabadi AM, Malcolm P.

Affiliation:

Department of Biomechanics and Center for Research in Human Movement Variability, University of Nebraska at Omaha, 6160 University Drive South, Omaha, NE 68182, USA

Corresponding Author:

Prokopios Antonellis

Department of Biomechanics and Center for Research in Human Movement Variability

University of Nebraska at Omaha

6160 University Drive

Omaha, NE 68182, USA.

Email: pantonellis@unomaha.edu

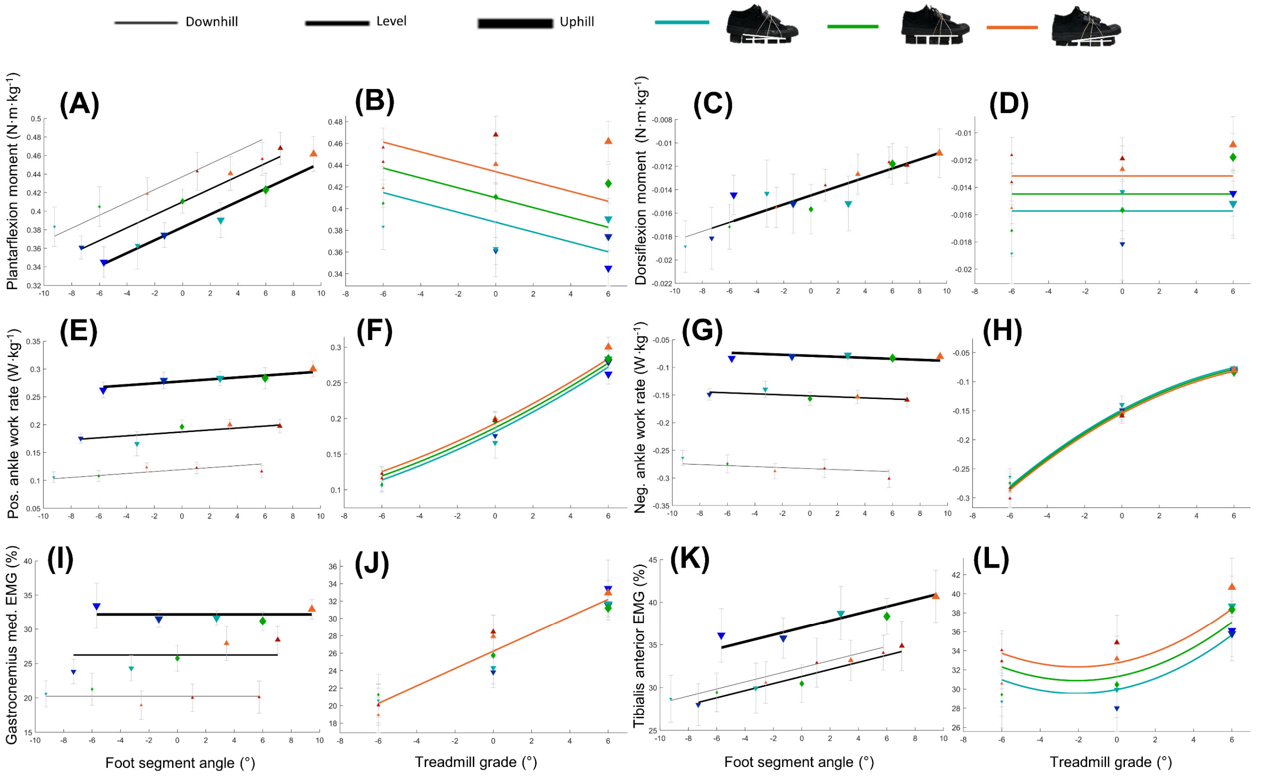

Figure S1. Ankle joint kinetic and muscle activity metrics. A, B: Average plantarflexion moment. C, D: Average dorsiflexion moment. E, F: Positive (pos.) ankle work rate. G, H: Negative (neg.) ankle work rate. I, J: Average gastrocnemius medialis (med.) EMG. K, L: Average tibialis anterior EMG. Panels A, C, E, G, I, and K show changes versus foot segment angle during downhill, level, and uphill walking. Black lines represent the formula from the linear mixed-effects model analysis evaluated over the tested range of foot segment angle at each treadmill grade. Panels B, D, F, H, J, and L show changes versus treadmill grade during walking in different shoe inclinations. Coloured lines represent the formula from the linear mixed-effects model analysis evaluated over the range of treadmill grades. Although five shoe inclinations were tested at each treadmill grade and were included in the evaluation of the statistical model, we only plotted the lines representing the evaluation of the linear mixed-effects model analysis for the three shoe inclinations that were tested on all three treadmill grades. Blue and cyan downward-pointing triangles represent mean values of conditions with downward shoe inclinations. Green diamonds represent mean values of conditions with level shoes. Orange and red upward-pointing triangles represent mean values of conditions with upward shoe inclinations. Error bars are s.e.m.

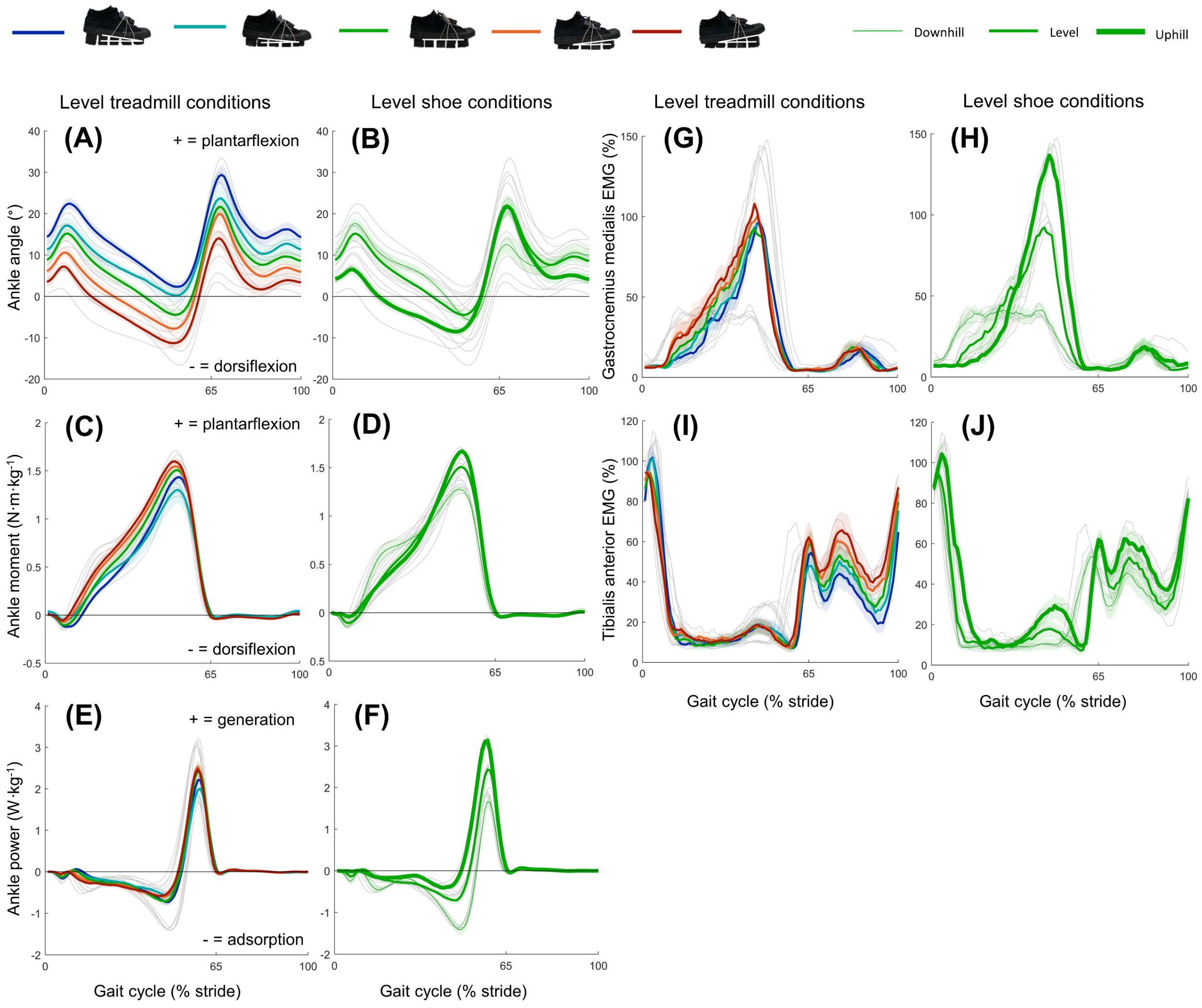

Figure S2. Ankle joint kinematic, kinetic and muscle activity time series. A, B: Ankle joint angle. C, D: Ankle joint moment. E, F: Ankle joint power. G, H: Gastrocnemius medialis EMG. I, J: Tibialis anterior EMG. Panels A, C, E, G, and I show the differences between shoe inclinations during walking on level treadmill represented by different coloured lines. Grey lines represent other than level treadmill grades. Panels B, D, F, H, and J show the differences between treadmill grades during walking with level shoes represented by different line thicknesses. Grey lines represent other shoe inclinations. All lines are means of all participants plotted versus stride time. Transparent bands are s.e.m.

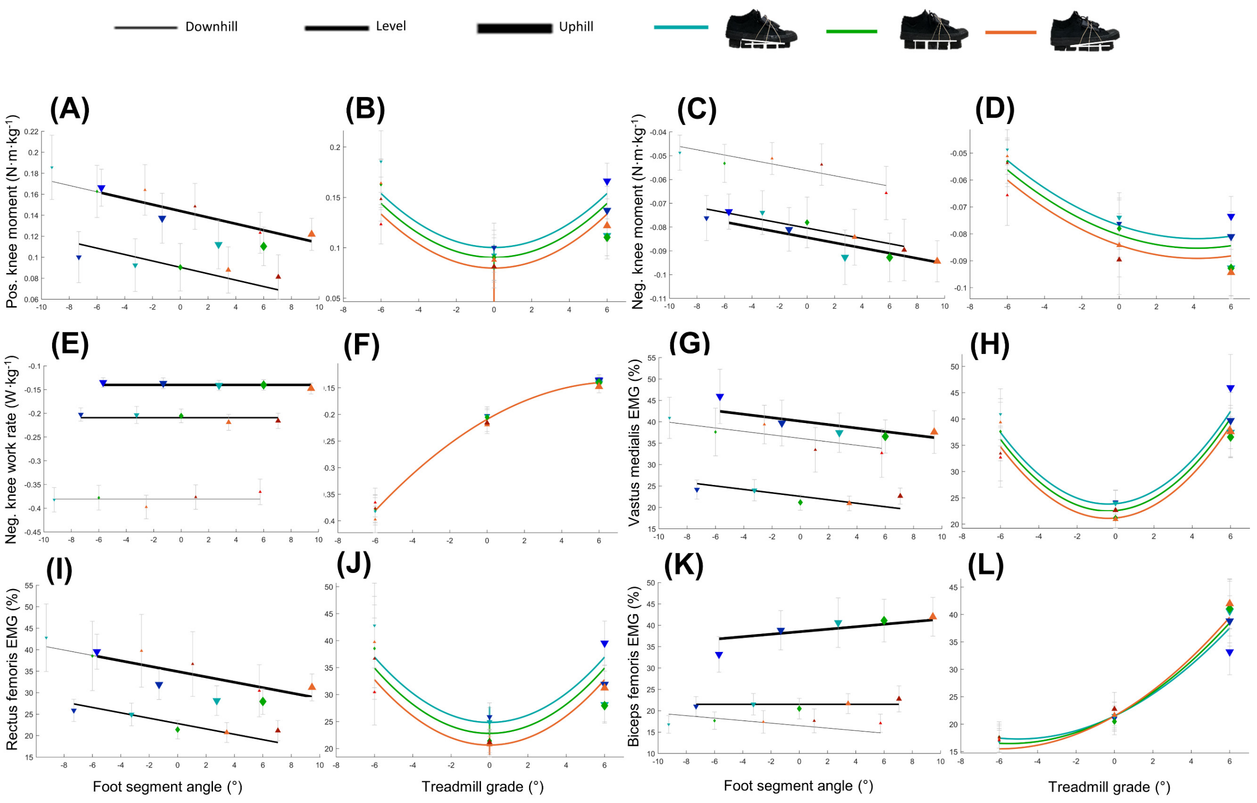

Figure S3. Knee joint kinetic and muscle activity metrics. A, B: Average positive (pos.) knee moment. C, D: Average negative (neg.) knee moment. E, F: Negative (neg.) knee work rate. G, H: Average vastus medialis EMG. I, J: Average rectus femoris EMG. K, L: Average bicep femoris EMG. Panels A, C, E, G, I, and K show changes versus foot segment angle during downhill, level, and uphill walking. Black lines represent the formula from the linear mixed-effects model analysis evaluated over the tested range of foot segment angle at each treadmill grade. Panels B, D, F, H, J, and L show changes versus treadmill grade during walking in different shoe inclinations. Coloured lines represent the formula from the linear mixed-effects model analysis evaluated over the range of treadmill grades. Although five shoe inclinations were tested at each treadmill grade and were included in the evaluation of the statistical model, we only plotted the lines representing the evaluation of the linear mixed-effects model analysis for the three shoe inclinations that were tested on all three treadmill grades. Blue and cyan downward-pointing triangles represent mean values of conditions with downward shoe inclinations. Green diamonds represent mean values of conditions with level shoes. Orange and red upward-pointing triangles represent mean values of conditions with upward shoe inclinations. Error bars are s.e.m.

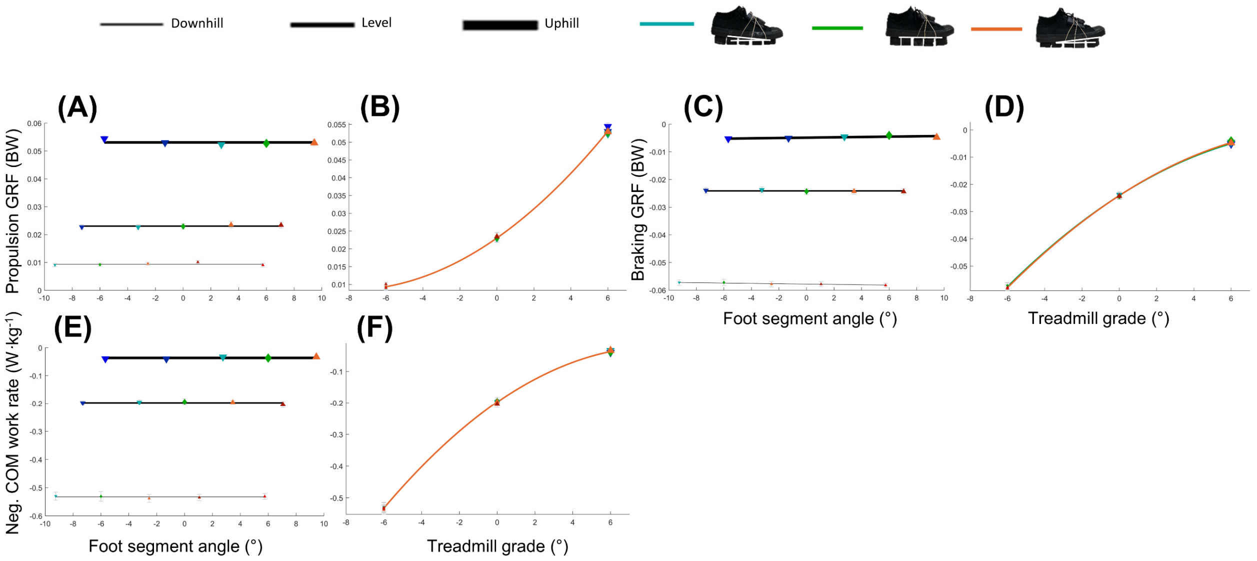

Figure S4. Ground reaction force and centre of mass metrics. A, B: Average propulsion GRF. C, D: Average braking GRF. E, F: Positive (pos.) COM work rate. G, H: Negative (neg.) COM work rate. Panels A, C, E, and G show changes versus foot segment angle during downhill, level, and uphill walking. Black lines represent the formula from the linear mixed-effects model analysis evaluated over the tested range of foot segment angle at each treadmill grade. Panels B, D, F, and H show changes versus treadmill grade during walking in different shoe inclinations. Coloured lines represent the formula from the linear mixed-effects model analysis evaluated over the range of treadmill grades. Although five shoe inclinations were tested at each treadmill grade and were included in the evaluation of the statistical model, we only plotted the lines representing the evaluation of the linear mixed-effects model analysis for the three shoe inclinations that were tested on all three treadmill grades. Blue and cyan downward-pointing triangles represent mean values of conditions with downward shoe inclinations. Green diamonds represent mean values of conditions with level shoes. Orange and red upward-pointing triangles represent mean values of conditions with upward shoe inclinations. Error bars are s.e.m.

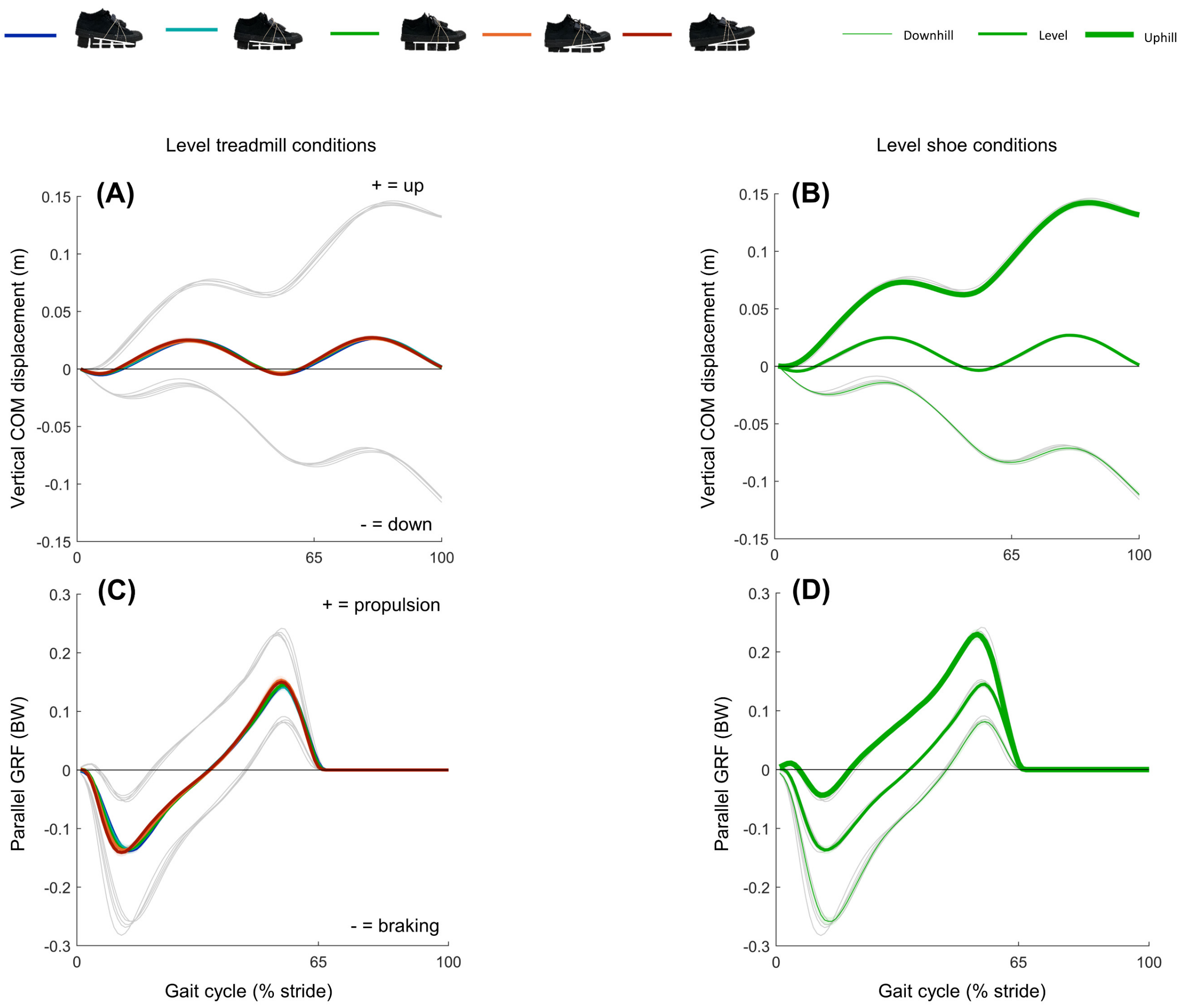

Figure S5. Ground reaction force and centre of mass time series. A, B: Vertical COM displacement. C, D: Component of the ground reaction force that is parallel to the treadmill. Panels A and C show the differences between shoe inclinations during walking on level treadmill represented by different coloured lines. Grey lines represent other than level treadmill grades. Panels B and D show the differences between treadmill grades during walking with level shoes represented by different line thicknesses. Grey lines represent other shoe inclinations. All lines are means of all participants plotted versus stride time. Transparent bands are s.e.m.

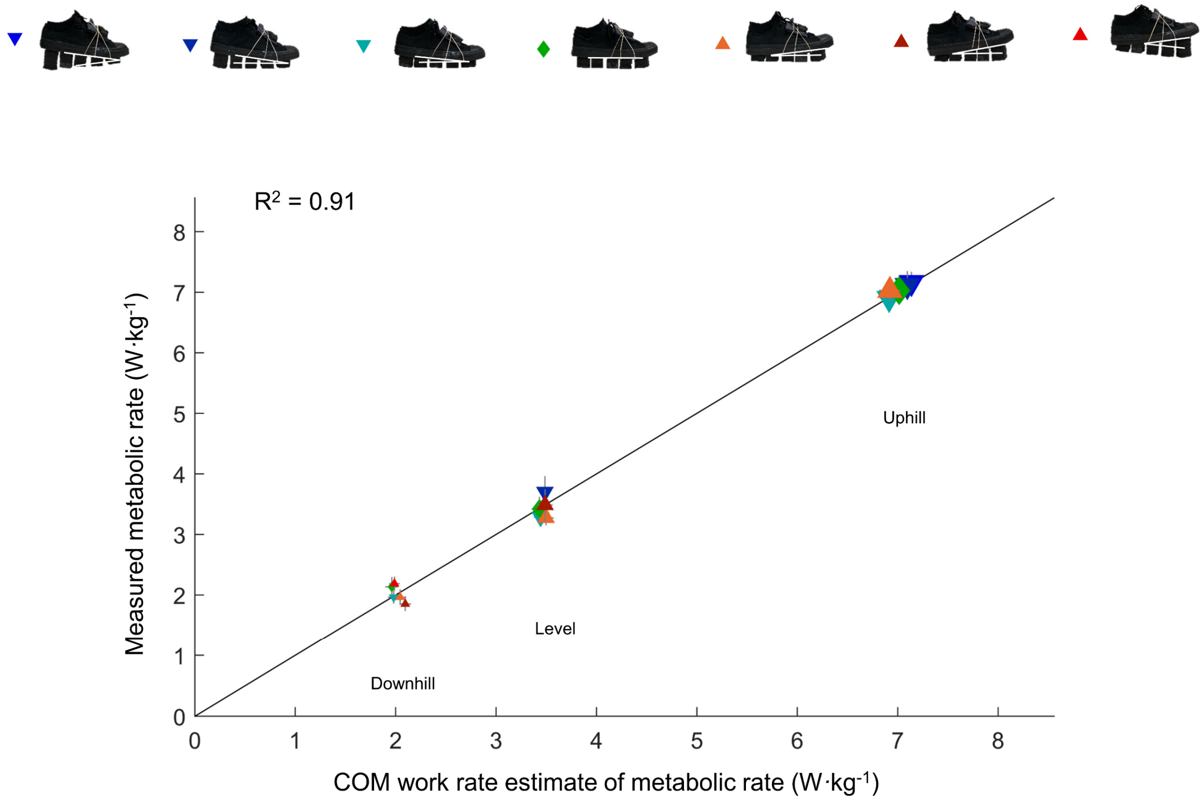

Figure S6. Relationship between centre of mass work rate and metabolic rate. To determine which parameters best explain the variance in metabolic rate, we also evaluated how different biomechanical parameters relate to changes in metabolic rate. We found one of the highest explained variances using only positive COM work rate ($P < 0.001$). This could be the result of the dominating effects of the uphill walking conditions on metabolic rate. Values on the X-axis in this plot represent the estimated metabolic rate based on best-fitting relationship between positive COM work rate and metabolic rate from linear mixed-effects model analysis (Metabolic rate ≈ 11.12 positive COM work rate + 1.40). R^2 value represents the coefficient of determination of measured metabolic rate versus estimated metabolic rate over all trials from all participants based on model from linear mixed-effects model analysis. Blue and cyan downward-pointing triangles represent mean values of conditions with downward shoe inclinations. Green diamonds represent mean values of conditions with level shoes. Orange and red upward-pointing triangles represent mean values of conditions with upward shoe inclinations. Error bars are s.e.m. The black line is the identity line.

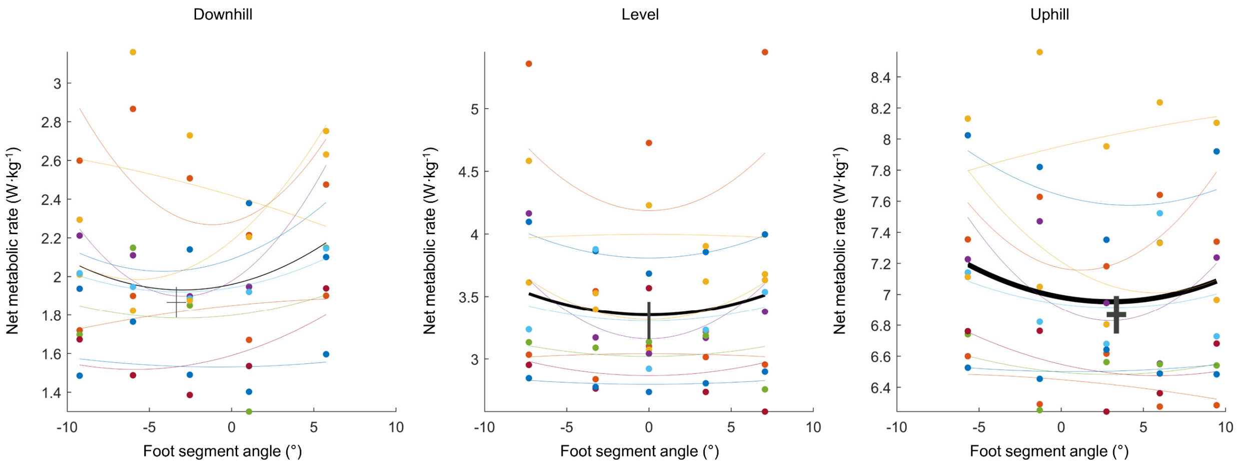

Figure S7. Individual participant responses of foot segment angle on metabolic rate during downhill, level, and uphill walking. Coloured lines show the individual trends (every colour is one participant). The thick black line shows the average trend. The dark grey crosses show the mean ± s.e.m of the locations of the minima.

Table S1. Effects of independent condition variables on net metabolic rate and biomechanical variables. Results of linear mixed-effects model analysis (random effect: participant; fixed effects: first and second order of foot segment angle and treadmill grade; outcome parameters: metabolic rate, ankle mechanics, COM and GRF mechanics). Values indicate resulting equation coefficients.

Variables	Intercept coefficient	Foot segment angle	Foot segment angle^2	Treadmill grade	Treadmill grade^2	Foot segment angle · Treadmill grade
Net metabolic rate ($W \cdot kg^{-1}$)	3.35**	NA	0.003*	0.41**	0.03**	-0.003*
Average plantarflexion moment ($N \cdot m \cdot kg^{-1}$)	0.40**	0.006**	NA	-0.004**	NA	NA
Average dorsiflexion moment ($N \cdot m \cdot kg^{-1}$)	-0.01**	0.0003**	NA	NA	NA	NA
Positive ankle work rate ($W \cdot kg^{-1}$)	0.18**	0.001**	NA	0.01**	0.0003*	NA
Negative ankle work rate ($W \cdot kg^{-1}$)	-0.15**	-0.0009*	NA	0.01*	-0.0008**	NA
Average soleus EMG (%)	26.93**	NA	0.03**	0.67**	0.08**	NA
Average gastrocnemius medialis (%)	26.21**	NA	NA	0.99**	NA	NA
Average tibialis anterior (%)	31.28**	0.41**	NA	0.39**	0.09**	NA
Positive COM work rate ($W \cdot kg^{-1}$)	0.18**	NA	NA	0.03**	0.002**	-0.0001*
Negative COM work rate ($W \cdot kg^{-1}$)	-0.19**	NA	NA	0.04**	-0.002**	NA
Average positive parallel GRF (BW)	0.02**	NA	NA	0.003**	0.0002**	NA
Average negative parallel GRF (BW)	-0.02**	NA	NA	0.004**	-0.0001**	0.00001*

*P < 0.05, **P < 0.01
NA = not applicable to final model due to non-significant contribution
BW, bodyweight
